# Benzodiazepine exposure during early pregnancy and risk of miscarriage: dose–response, half-life classification, and individual agents

**DOI:** 10.1007/s00737-026-01720-3

**Published:** 2026-05-23

**Authors:** Tomofumi Ishikawa, Takamasa Sakai, Noriyuki Iwama, Ryo Obara, Kei Morishita, Motohiko Adomi, Tadaharu Kunitoki, Aoi Noda, Mami Ishikuro, Saya Kikuchi, Natsuko Kobayashi, Hiroaki Tomita, Masatoshi Saito, Hidekazu Nishigori, Shinichi Kuriyama, Nariyasu Mano, Taku Obara

**Affiliations:** 1 Laboratory of Biomolecule and Pathophysiological Chemistry, Graduate School of Pharmaceutical Sciences, Tohoku University, Sendai, Japan; 2 Drug Informatics, Faculty of Pharmacy, Meijo University, Nagoya, Japan; 3 Center for Maternal and Perinatal Medicine, Tohoku University Hospital, Sendai, Japan; 4 Division of Molecular Epidemiology, Graduate School of Medicine, Tohoku University, Sendai, Japan; 5 Department of Epidemiology, Harvard T.H. Chan School of Public Health, Boston, United States; 6 Division of Clinical Pharmacology and Therapeutics, Graduate School of Pharmaceutical Sciences, Tohoku University, Sendai, Japan; 7 Department of Preventive Medicine and Epidemiology, Tohoku Medical Megabank Organization, Tohoku University, Sendai, Japan; 8 Department of Pharmaceutical Sciences, Tohoku University Hospital, Sendai, Japan; 9 Department of Psychiatry, Graduate School of Medicine, Tohoku University, Sendai, Japan; 10 Department of Obstetrics and Gynecology, Graduate School of Medicine, Tohoku University, Sendai, Japan; 11 Department of Development and Environmental Medicine, Fukushima Medical Center for Children and Women, Graduate School of Medicine, Fukushima Medical University, Fukushima, Japan; 12 International Research Institute of Disaster Science, Tohoku University, Sendai, Japan

**Keywords:** Anxiety, Benzodiazepine, Miscarriage, Pregnancy, Sleep disorder

## Abstract

**Purpose:**

To evaluate the association between benzodiazepine exposure during early pregnancy and the risk of miscarriage, with a focus on dose–response, half-life, and individual agents.

**Methods:**

This nested case–control study was conducted using a large Japanese administrative database. Cases were defined as pregnancies ending in miscarriage between 2005 and 2023. Controls were randomly selected through risk-set sampling with replacement and matched in a 3:1 ratio. Conditional logistic regression, adjusted for confounders, was used to assess the associations.

**Results:**

Overall, 63,482 cases were matched with 190,383 controls, with a mean age of 33.2 years. The prevalence of benzodiazepine exposure was 1.7% and 1.2% among cases and controls, respectively. Etizolam (0.3%) had the highest prevalence, followed by alprazolam (0.3%) and brotizolam (0.2%) in both groups. Adjusted odds ratio (OR) for benzodiazepine exposure during early pregnancy was 1.241 (95% confidence interval: 1.136–1.357). Adjusted ORs for high-dose (1.212 [1.050–1.400]) and medium-dose (1.189 [1.037–1.363]) exposure were numerically higher than that for low-dose exposure (1.111 [0.934–1.322]). Adjusted OR for long-acting benzodiazepines (1.308 [1.124–1.522]) was numerically higher than those for intermediate- (1.132 [0.999–1.284]) and short-acting (1.157 [1.030–1.300]) agents. Adjusted ORs for etizolam, alprazolam, and brotizolam were 0.929 (0.782–1.103), 0.853 (0.707–1.030), and 1.454 (1.180–1.791), respectively.

**Conclusions:**

Benzodiazepine exposure during early pregnancy was modestly associated with an increased risk of miscarriage. Although associations tended to be stronger with higher dose levels and longer half-life categories, estimates across subgroups lacked precision and should be interpreted with caution.

**Supplementary Information:**

The online version contains supplementary material available at https://doi.org/10.1007/s00737-026-01720-3.

## Article Highlights

This study evaluated the risk of miscarriage associated with benzodiazepine use in early pregnancy.

Benzodiazepine use in early pregnancy was associated with a modestly increased risk of miscarriage.

Higher dose levels and longer-acting agents showed numerically higher estimates, although differences lack precision.

Results varied across sensitivity and subgroup analyses, and should be interpreted cautiously.

Japan-specific agents (e.g., etizolam) were evaluated to inform local prescribing decisions.

**Supplementary Information:**

The online version contains supplementary material available at https://doi.org/10.1007/s00737-026-01720-3.

## Introduction

Psychiatric and sleep disorders are common among pregnant women (Gentile 2014). Benzodiazepines are frequently prescribed during pregnancy to manage these conditions (Bais et al. 2020; Japanese Society of Perinatal Mental Health 2023; Shimoya et al. 2022). However, their use during pregnancy raises notable concerns, as they can cross the placenta and accumulate in embryonic and fetal tissues (Kanto 1982; Mandelli et al. 1975), potentially impacting fetal development (Wu et al. 2024).

Benzodiazepines may increase the risk of miscarriage owing to their potential effects on fetal development. Several studies have, therefore, evaluated the risk of miscarriage associated with benzodiazepine use. Meta-analyses (Grigoriadis et al. 2020; National Institute for Health and Care Excellence 2018) and observational studies (Meng et al. 2023; Sheehy et al. 2019) have reported an association between benzodiazepine exposure and miscarriage, although these associations may have been confounded by indications (Andrade 2019). Most previous studies have assessed benzodiazepines as a drug class, with limited evidence regarding their dose, half-life categories, or individual agents. Recent observational studies have examined the risk of miscarriage associated with specific benzodiazepines, including alprazolam, clonazepam, diazepam, lorazepam, and oxazepam (Meng et al. 2023; Sheehy et al. 2019). However, benzodiazepine use patterns vary by region. For instance, although oxazepam and temazepam are commonly used elsewhere (Bais et al. 2020), they are unavailable in Japan, where brotizolam, etizolam, and flunitrazepam are frequently prescribed (Inada et al. 2021). Therefore, further research is needed to clarify the association between prenatal benzodiazepine exposure and miscarriage risk and to assess the applicability of existing evidence across diverse populations.

Health administrative databases are valuable resources for carrying out pharmacoepidemiological evaluations of drug exposure and associated risks during pregnancy (Huybrechts et al. 2019). This nested case–control study aimed to evaluate the association between benzodiazepine exposure during early pregnancy and miscarriage risk, and to examine potential differences by drug class, dose levels, half-life categories, and several major individual benzodiazepines, using data from a large Japanese health administrative database.

## Methods

### Data source

Data were extracted from a large administrative claims database from JMDC Inc. (Tokyo, Japan) (Barberio et al. 2024; Nagai et al. 2021). The database contains inpatient, outpatient, and pharmacy claims received from insurers. Claims included diagnoses coded using the International Classification of Diseases, 10th Revision (ICD-10), surgical and medical procedures coded using standardized procedure codes, and dispensed medications classified under the World Health Organization (WHO)-Anatomical Therapeutic Chemical (ATC) Classification and other codes. Information on the year and month was available for all claims. Diagnosis dates are generally recorded except in rare cases (e.g., dates are not mandatory when a condition resolves or results in death within the same month). Implementation and dispensing dates are available for nearly all procedures and dispensations. All data are retained even when patients are transferred between hospitals or when they use multiple facilities. Previous studies have shown that the characteristics of individuals of reproductive age in the JMDC database are comparable to those of their counterparts in the national database (Barberio et al. 2024; Nagai et al. 2021). Standardized disease classification and anonymous record linkages were applied (Nagai et al. 2021).

This study was approved by the Institutional Review Board of Tohoku University School of Medicine on April 23, 2025 (registration number: 2025–1-087), and the requirement for informed consent was waived.

### Source population

**Fig. 1:**
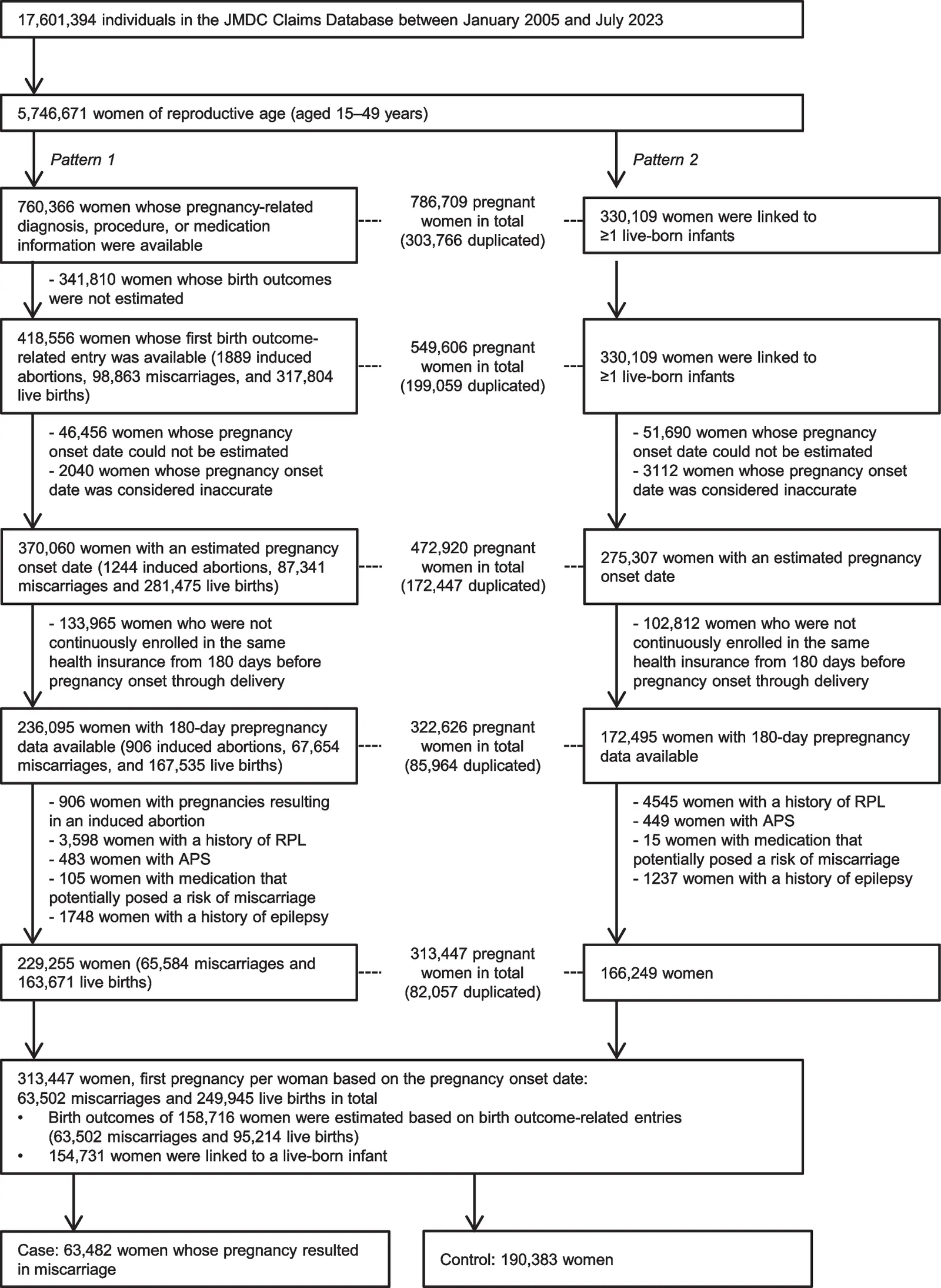
Flowchart illustrating case–control selection. Abbreviations: APS, antiphospholipid syndrome; RPL, recurrent pregnancy loss

The dataset available on March 12, 2024, included 17,601,394 individuals insured between January 2005 and July 2023 (Fig. 1). Women aged 15–49 years who met the following eligibility criteria were included: those whose pregnancy, birth outcome, and pregnancy period (i.e., dates of onset and end) could be estimated and those who were continuously enrolled in the database from 180 days before pregnancy onset until the end of pregnancy. Only the first pregnancy per woman (the one with the earliest onset date) recorded in the database was included. Given that the pregnancy onset date was estimated using a previously developed algorithm (Ishikawa et al. 2018), pregnancies with a gestational age (GA) at birth that was considered implausible, i.e., GA shorter than 4 weeks or longer than 22 weeks for miscarriage and induced abortion, and GA shorter than 22 weeks or longer than 42 weeks for live birth, were excluded. Women whose pregnancy ended in an induced abortion, those with a history of recurrent pregnancy loss, those diagnosed with antiphospholipid syndrome, and those who had been dispensed with medications potentially associated with miscarriage risk (Table S1) were also excluded. Additionally, women with a history of epilepsy (Table S1) were excluded because epilepsy may be teratogenic (Oliveira and Fett-Conte 2013), and benzodiazepines are commonly prescribed for epilepsy, and our focus was on benzodiazepine use for anxiety and sleep disorders.

### Estimation of pregnancy and birth outcomes

Information on pregnancy, including pregnancy onset, end, and outcomes, is unavailable in Japanese claims data because prenatal care is covered by a separate government-subsidized program (Barberio et al. 2024). Applying the methods used in other countries, based on the timing of prenatal care (Margulis et al. 2015), to the Japanese claims data can be challenging. Therefore, pregnancy and outcomes were estimated using previously reported algorithms developed in Japan (Committee on Obstetric Practice et al. 2017; Ishikawa et al. 2018, 2019, 2023, 2024, 2025; Japan Society of Obstetrics and Gynecology and Japan Association of Obstetricians and Gynecologists 2023; Tajima et al. 2022a, b), the protocol of which is detailed in the Supplementary Appendix, Figur S1, and Tables S2 and S3.

### Cases of miscarriage and selection of controls

A case–control design is suitable for studying triggers of an acute event by flexibly modeling the exposure window at varying proximities to the event of interest (Schneeweiss et al. 2019). Cases were defined as pregnancies ending in miscarriage between the beginning of the 4th week and 22nd week of gestation (i.e., week 4, day 0 to week 21, day 6). The date of miscarriage was considered the index date, and the follow-up period was defined as the period from pregnancy onset to the index date. Controls were randomly selected from the entire cohort of pregnancies by risk-set sampling with replacement and were individually matched to the cases at a 3:1 ratio. For this purpose, all cohort members still at risk within the follow-up period and with the same age at pregnancy onset (± 1 year), GA (in days), and year of pregnancy onset were selected as cases. Potential controls within the risk sets were assigned an index date corresponding to the same follow-up duration as the cases (i.e., the index date for the controls was their pregnancy onset date plus the matched case follow-up duration in days). A sampling method with replacement was used for control selection. Thus, a control could be matched to multiple cases, and a case could serve as a control before becoming a case. This process ensures that the odds ratio (OR) was an unbiased estimator of relative risk, approximating its true value (Kitchin et al. 2022; Vandenbroucke and Pearce 2012).

### Drug exposure

Benzodiazepines were identified using the WHO-ATC codes or alternate entries (Table S4). Non-oral and oral benzodiazepines, typically indicated for epilepsy, were not included in the scope of this study (Table S4). Based on their pharmacological half-lives, benzodiazepines were classified as short-, intermediate-, or long-acting (Table S4). To evaluate a dose-dependent effect, dose equivalence was calculated using diazepam equivalents (Inada and Inagaki 2015), categorized as low (≤ 5 mg/day as diazepam equivalence), medium (> 5 mg and < 15 mg), and high (≥ 15 mg), based on the mean daily dose derived from the cumulative diazepam-equivalent dose divided by the cumulative duration of exposure. As information on single or daily doses of as-needed medications was unavailable, only scheduled medications were included in the dose-dependent evaluation. Pregnancy was considered exposed if benzodiazepines were dispensed to the mother from the onset of pregnancy until the day prior to the index date. In the main analysis, a pregnancy was considered exposed if the days of supply overlapped the window between pregnancy onset and the index date. In a sensitivity analysis, only those with one or more dispensations during the window were considered as exposed (Huybrechts et al. 2019). Inpatient and Diagnosis Procedure Combination claims were considered inpatient, whereas outpatient and dispensing claims were considered as outpatient. The dispensing date was preferentially used to estimate the timing of exposure; however, if the dispensing date was unavailable, the 15th day of the month and year of each claim was assigned.

### Potential confounders

Potential confounders were selected based on known risk factors for miscarriage (Japan Society of Obstetrics and Gynecology and Japan Association of Obstetricians and Gynecologists 2023; Quenby et al. 2021), general markers of illness burden (Huybrechts et al. 2019), proxies for disease severity or benzodiazepine exposure (Table S1). In general, these data were obtained from the pre-exposure covariate assessment window to avoid adjusting for causal intermediates (Schneeweiss and Suissa 2019).

### Analysis

The characteristics of the cases and controls as well as the prevalence of benzodiazepine exposure, were described. The ORs and 95% confidence intervals (CIs) were calculated using conditional logistic regression, both unadjusted and adjusted for confounders, for any benzodiazepine dose level, half-life category, and several major individual agents (i.e., those with ≥ 100 exposed in total, including diazepam, loflazepate, alprazolam, bromazepam, flunitrazepam, lorazepam, nitrazepam, brotizolam, clotiazepam, etizolam and triazolam). Several sensitivity analyses were conducted. See the Supplementary Appendix and Table S5 for additional details regarding the sensitivity analyses (Sundermann et al. 2019; van Gelder et al. 2023).

All data were analyzed using SAS version 9.4 (SAS Institute Inc., Cary, NC, USA). The interpretations were based on point estimates and 95% CIs of the adjusted ORs. To avoid missing potential safety signals, no adjustments were made for multiple comparisons.

## Results

A total of 313,447 women met all the eligibility criteria, including 63,502 and 249,945 women whose first pregnancies in the database ended in miscarriages and live births, respectively (Fig. 1). Among these, 63,482 cases were matched to 190,383 controls, with three controls identified for 63,438 cases. Dispensing dates were available for 98.1% of benzodiazepine dispensation records and were used to estimate the exposure timing. For the remaining 1.9%, the 15th day of the recorded month and year for each claim was assigned.

The baseline characteristics are summarized in Table 1. The mean maternal age was 33.2 years in both groups. The prevalence of benzodiazepine exposure was 1.7% (1076/63,482) and 1.2% (2281/190,383) in the case and control groups, respectively (Table 2). Scheduled and as-needed benzodiazepine dispensations were recorded for 925 (1.5%) and 260 (0.4%) in the case group, and 1973 (1.0%) and 565 (0.3%) in the control group, respectively. For scheduled benzodiazepine dispensation, the mean (SD) duration of estimated exposure was 41.8 (31.2) and 45.8 (26.3) days in the case and control groups, respectively. Outpatient and inpatient dispensations were recorded for 948 (1.5%) and 133 (0.2%) in the case group and for 2263 (1.2%) and 30 (0.0%) in the control group, respectively. The prevalence of high-, medium-, and low-dose benzodiazepine exposure was 0.5%, 0.6%, and 0.3% in the case group and 0.4%, 0.4%, and 0.3% in the control group, respectively (Table 2). The prevalence of short-acting benzodiazepines (0.8% among cases and 0.6% among controls) was higher than those of long-acting (0.5% and 0.3%) or intermediate-acting (0.7% and 0.5%) agents (Table 2). Etizolam (0.3% in both groups) had the highest prevalence among individual benzodiazepines, followed by alprazolam (0.3%) and brotizolam (0.2%).

**Table 1:** Baseline characteristics of case–control groups

		Cases (miscarriages) *n* = 63,482	Controls *n* = 190,383
Age at pregnancy onset (year), mean (SD) or n (%)		33.2 (5.6)	33.2 (5.5)
≤ 24		3973 (6.3)	11472 (6.0)
25–29		12899 (20.3)	39012 (20.5)
30–34		19522 (30.8)	59228 (31.1)
≥ 35		27088 (42.7)	80671 (42.4)
Year of pregnancy onset, n (%)			
2005–2009		1735 (2.7)	5188 (2.7)
2011–2013		4941 (7.8)	14819 (7.8)
2014–2016		11170 (17.6)	33506 (17.6)
2017–2019		20538 (32.4)	61610 (32.4)
2020–2023		25098 (39.5)	75260 (39.5)
Main indications of benzodiazepines, n (%)		3227 (5.1)	8057 (4.2)
Anxiety disorder		1654 (2.6)	4290 (2.3)
Sleep disorder		2133 (3.4)	5323 (2.8)
Other psychiatric disorders, n (%)		1591 (2.5)	4345 (2.3)
Schizophrenia		321 (0.5)	870 (0.5)
Manic episode		24 (0.0)	66 (0.0)
Bipolar affective disorder		222 (0.3)	625 (0.3)
Depressive disorder		1375 (2.2)	3764 (2.0)
Uterine diseases, n (%)		8432 (13.3)	24244 (12.7)
Endometriosis		5263 (8.3)	14917 (7.8)
Polyp of corpus uteri		2297 (3.6)	7235 (3.8)
Polyp of cervix uteri		1570 (2.5)	4480 (2.4)
Cervix carcinoma		76 (0.1)	263 (0.1)
Corpus uteri carcinoma		58 (0.1)	113 (0.1)
Congenital uterus and cervix malformations		170 (0.3)	420 (0.2)
Other maternal comorbidities, n (%)		6673 (10.5)	21110 (11.1)
Polycystic ovary syndrome		1872 (2.9)	5397 (2.8)
Diabetes		1176 (1.9)	3969 (2.1)
Obesity		181 (0.3)	606 (0.3)
Thyroid disorders		4286 (6.8)	13926 (7.3)
Dependence, n (%)		82 (0.1)	226 (0.1)
Alcohol		21 (0.0)	63 (0.0)
Tobacco		62 (0.1)	163 (0.1)
No. of mental health medications dispensed in the 6 months prior to pregnancy onset, mean (SD) or n (%)		0.1 (0.4)	0.1 (0.3)
0		60262 (94.9)	180697 (94.9)
1–2		2940 (4.6)	8963 (4.7)
≥ 3		280 (0.4)	723 (0.4)
No. of other medications dispensed in the 6 months prior to pregnancy onset, mean (SD)		5.8 (6.4)	5.8 (6.4)
No. of diagnoses in the 6 months prior to pregnancy onset, mean (SD)		7.4 (4.1)	6.0 (4.1)

Abbreviation: *SD* Standard deviation

**Table 2:** Benzodiazepines dispensed from the onset of pregnancy through the index date

	Cases (miscarriages) *n* = 63,482	Controls *n* = 190,383
Any benzodiazepine	1076 (1.7)	2281 (1.2)
Classified by dose level		
High-dose	344 (0.5)	700 (0.4)
Medium-dose	366 (0.6)	788 (0.4)
Low-dose	215 (0.3)	485 (0.3)
Classified by half-life and individual benzodiazepine		
Long-acting	306 (0.5)	577 (0.3)
Chlordiazepoxide	7 (0.0)	23 (0.0)
Clonazepam	32 (0.1)	63 (0.0)
Clorazepate	3 (0.0)	3 (0.0)
Cloxazolam	24 (0.0)	52 (0.0)
Diazepam	103 (0.2)	113 (0.1)
Fludiazepam	2 (0.0)	6 (0.0)
Flurazepam	1 (0.0)	2 (0.0)
Loflazepate	129 (0.2)	295 (0.2)
Medazepam	1 (0.0)	9 (0.0)
Mexazolam	2 (0.0)	3 (0.0)
Oxazolam	1 (0.0)	6 (0.0)
Prazepam	0 (0.0)	0 (0.0)
Quazepam	5 (0.0)	7 (0.0)
Intermediate-acting	457 (0.7)	1024 (0.5)
Alprazolam	169 (0.3)	484 (0.3)
Bromazepam	67 (0.1)	150 (0.1)
Estazolam	14 (0.0)	18 (0.0)
Flunitrazepam	84 (0.1)	188 (0.1)
Lorazepam	122 (0.2)	223 (0.1)
Nimetazepam	2 (0.0)	0 (0.0)
Nitrazepam	55 (0.1)	71 (0.0)
Short-acting	530 (0.8)	1143 (0.6)
Brotizolam	158 (0.2)	301 (0.2)
Clotiazepam	106 (0.2)	249 (0.1)
Etizolam	215 (0.3)	532 (0.3)
Flutazolam	1 (0.0)	4 (0.0)
Lormetazepam	14 (0.0)	42 (0.0)
Rilmazafone	34 (0.1)	17 (0.0)
Tofisopam	15 (0.0)	38 (0.0)
Triazolam	47 (0.1)	101 (0.1)

Crude and adjusted ORs for benzodiazepine exposure were 1.421 (95% CI, 1.321–1.529) and 1.241 (1.136–1.357), respectively (Table 3). The adjusted ORs for high-dose (1.212 [95% CI: 1.050–1.400]) and medium-dose (1.189 [1.037–1.363]) exposure were numerically higher than that for low-dose exposure (1.111 [0.934–1.322]) (Table 3). The adjusted OR for long-acting benzodiazepines (1.308 [1.124–1.522]) was numerically higher than those for intermediate- (1.132 [0.999–1.284]) and short-acting (1.157 [1.030–1.300]) agents. For individual drugs, the adjusted ORs were 0.929 (0.782–1.103) for etizolam, 0.853 (0.707–1.030) for alprazolam, and 1.454 (1.180–1.791) for brotizolam. Additionally, the adjusted ORs were 2.096 (1.580–2.780) for diazepam, 1.327 (1.046–1.682) for lorazepam, and 2.134 (1.461–3.116) for nitrazepam.

**Table 3:** Benzodiazepines and the risk of miscarriage

	Crude OR (95% CI)	Adjusted OR^a^ (95% CI)
Any benzodiazepine	1.421 (1.321, 1.529)	1.241 (1.136, 1.357)
Classified by dose level		
High-dose	1.476 (1.297, 1.680)	1.212 (1.050, 1.400)
Medium-dose	1.394 (1.231, 1.578)	1.189 (1.037, 1.363)
Low-dose	1.332 (1.134, 1.565)	1.111 (0.934, 1.322)
Classified by half-life and by individual benzodiazepine		
Long-acting	1.592 (1.386, 1.828)	1.308 (1.124, 1.522)
Diazepam	2.735 (2.094, 3.571)	2.096 (1.580, 2.780)
Loflazepate	1.312 (1.067, 1.613)	1.115 (0.894, 1.390)
Intermediate-acting	1.340 (1.200, 1.497)	1.132 (0.999, 1.284)
Alprazolam	1.048 (0.879, 1.248)	0.853 (0.707, 1.030)
Bromazepam	1.340 (1.005, 1.787)	1.060 (0.781, 1.438)
Flunitrazepam	1.341 (1.037, 1.736)	1.224 (0.931, 1.609)
Lorazepam	1.641 (1.316, 2.047)	1.327 (1.046, 1.682)
Nitrazepam	2.324 (1.634, 3.305)	2.134 (1.461, 3.116)
Short-acting	1.394 (1.257, 1.545)	1.157 (1.030, 1.300)
Brotizolam	1.572 (1.297, 1.906)	1.454 (1.180, 1.791)
Clotiazepam	1.278 (1.018, 1.605)	1.068 (0.838, 1.360)
Etizolam	1.214 (1.035, 1.422)	0.929 (0.782, 1.103)
Triazolam	1.396 (0.988, 1.973)	1.133 (0.789, 1.629)

Abbreviations: *CI* Confidence interval, *OR* Odds ratio

^a^Adjusted for the main indications for benzodiazepines (anxiety disorder or sleep disorder); other psychiatric disorders (schizophrenia, manic episode, bipolar affective disorder, or depressive disorder); uterine diseases (endometriosis, polyp of corpus uteri or cervix uteri, cervix or corpus uteri carcinoma, or congenital uterus and cervix malformations); other maternal comorbidities (polycystic ovary syndrome, diabetes, obesity, or thyroid disorders); alcohol or tobacco dependence; the number of mental health medications dispensed in the 6 months prior to pregnancy onset; the number of other medications dispensed in the 6 months prior to pregnancy onset; and the number of diagnoses in the 6 months prior to pregnancy onset

In the first five sensitivity analyses, the findings were not consistently in agreement with those of the main analysis (Tables S7–S11). Adjusted ORs for benzodiazepines ranged from 0.984 to 1.263. Dose-dependent trends were inconsistent across some sensitivity analyses, with adjusted ORs as follows: high-dose, 0.969–1.182; medium-dose, 0.936–1.404; and low-dose, 1.069–1.151. The adjusted ORs for long-, intermediate-, and short-acting benzodiazepines were 1.017–1.421, 1.024–1.141, and 0.960–1.220, respectively. In the sixth sensitivity analysis, limited to women diagnosed with anxiety or sleep disorders, 2564 cases were matched with 7624 controls; the adjusted OR for any benzodiazepine was 1.118 (95% CI, 1.001–1.248) (Table 4). Dose-dependent trends were not observed, and adjusted ORs were comparable across the half-life categories (Table 4).

**Table 4:** Benzodiazepines and the risk of miscarriage – sensitivity analysis of women diagnosed with anxiety or sleep disorders

	Crude OR (95% CI)	Adjusted OR^a^ (95% CI)
Any benzodiazepine	1.161 (1.048, 1.286)	1.118 (1.001, 1.248)
Classified by dose level		
High-dose	1.236 (1.021 1.496)	1.174 (0.965, 1.430)
Medium-dose	0.913 (0.716,1.164)	0.867 (0.676, 1.112)
Low-dose	1.214 (1.063, 1.386)	1.176 (1.024, 1.351)
Classified by half-life and individual benzodiazepine		
Long-acting	1.140 (0.951, 1.366)	1.084 (0.899, 1.307)
Intermediate-acting	1.217 (1.063, 1.393)	1.175 (1.018, 1.356)
Short-acting	1.118 (0.980, 1.275)	1.076 (0.938, 1.234)

Abbreviations: *CI* Confidence interval, *OR* Odds ratio

^a^Adjusted for other psychiatric disorders (schizophrenia, manic episode, bipolar affective disorder, or depressive disorder); uterine diseases (endometriosis, polyp of corpus uteri or cervix uteri, cervix or corpus uteri carcinoma, or congenital uterus and cervix malformations); other maternal comorbidities (polycystic ovary syndrome, diabetes, obesity, or thyroid disorders); alcohol or tobacco dependence; the number of mental health medications dispensed in the 6 months prior to pregnancy onset; the number of other medications dispensed in the 6 months prior to pregnancy onset; and the number of diagnoses in the 6 months prior to pregnancy onset

## Discussion

We found that benzodiazepine exposure in early pregnancy is modestly associated with miscarriage risk. Nevertheless, the results of some of the sensitivity analyses yielded slightly lower ORs than those of the main analysis. Point estimates were numerically higher for certain dose levels and half-life categories; however, CIs overlapped across categories, indicating limited precision for between-category comparisons. Furthermore, we evaluated individual benzodiazepines, which may help bridge the knowledge gap in clinical practice.

According to the clinical guidance of the National Institute for Health and Care Excellence, some evidence suggests an increased miscarriage risk based on a meta-analysis integrating three studies (OR 1.83, 95% CI: 1.19–2.82). Another meta-analysis of five pooled studies reported a significant association between benzodiazepine exposure and miscarriage (OR 1.86, 95% CI: 1.43–2.42), although heterogeneity was noted (National Institute for Health and Care Excellence 2018). These meta-analyses evaluated benzodiazepines as a drug class. Recent observational studies have reported an increased miscarriage risk associated with benzodiazepine use during pregnancy. A Canadian nested case–control study reported that benzodiazepine exposure in early pregnancy was associated with an increased miscarriage risk (adjusted OR 1.85, 95% CI: 1.61–2.12) (Sheehy et al. 2019). The association between benzodiazepines and miscarriage was strengthened with increasing diazepam-equivalent defined daily dose (DDD): < 5 mg: adjusted OR 1.73 (95% CI, 1.44–2.08); 6‒20 mg: adjusted OR 1.96 (95% CI, 1.59–2.43); and > 20 mg: adjusted OR 2.55 (95% CI, 1.08–6.01; *P* < 0.01). The risk was similar among pregnancies exposed to short-acting (half-life ≤ 24 h) benzodiazepines (adjusted OR 1.81, 95% CI: 1.55–2.12) and long-acting (half-life > 24 h) agents (adjusted OR 1.73, 1.31–2.28) during early pregnancy. All benzodiazepines examined in that study (alprazolam, bromazepam, clonazepam, diazepam, flurazepam, lorazepam, oxazepam, temazepam, and triazolam) were independently associated with an increased miscarriage risk (adjusted OR 1.13–3.43). A case-time-control study from Taiwan reported that benzodiazepine use during pregnancy was associated with an increased miscarriage risk (OR 1.69, 95% CI: 1.52–1.87) (Meng et al. 2023). Both long-acting (half-life > 24 h) and short-acting (half-life ≤ 24 h) benzodiazepines revealed an increased miscarriage risk, with case-time-control ORs of 1.67 (1.44–1.93) and 1.66 (1.47–1.87), respectively. Additionally, a dose–response association was noted, with ORs increasing from 1.61 (1.43–1.82) for low-dose exposure (DDD < 1.0) to 1.86 (1.53–2.25) for high-dose exposure (DDD ≥ 1.0); an increased miscarriage risk was also associated with each commonly used individual benzodiazepine (alprazolam, diazepam, lorazepam, oxazolam, and fludiazepam), with case-time-control ORs of 1.39 (1.17–1.66) for alprazolam to 2.52 (1.89–3.36) for fludiazepam.

Compared with prior observational studies, the magnitude of association observed in our study was smaller (adjusted OR 1.241, 95% CI 1.136–1.357), although directionally consistent with the overall literature supporting an increased risk associated with benzodiazepine exposure in early pregnancy. In our previous study, use of benzodiazepines as a positive control yielded an adjusted OR of 1.431 (95% CI: 1.303–1.573); however, major indications, such as anxiety and sleep disorders, were not considered as covariates (Ishikawa et al. 2024). In this study, after adjusting for these covariates, the adjusted OR decreased to 1.241. Some sensitivity analyses yielded lower ORs than the main analysis, suggesting that the magnitude of association may be sensitive to analytic assumptions. Although associations appeared stronger for higher dose levels and longer half-life categories, indicating limited precision for between-category comparisons. Potential explanations for inconsistencies between this and previous studies and across analyses within our study include reduced statistical power, exposure misclassification related to exposure timing definitions, and residual confounding despite adjustment. The main analysis is considered the most appropriate because it incorporates days of supply and aligns exposure assessment with the etiologically relevant window from pregnancy onset to the index date. Nevertheless, as each sensitivity analysis reflects a plausible alternative assumption, the overall interpretation should consider the range of results observed across analytic approaches. Of note, ORs overestimate the magnitude of risk compared with relative risk values (Doi et al. 2022). The evidence may also suggest that the true association between benzodiazepine exposure and miscarriage may be modest under the dosage and prescription patterns of benzodiazepines in Japan. The adjusted OR for brotizolam was relatively high despite being a short-acting benzodiazepine, likely because most diazepam-equivalent doses of brotizolam were categorized as high.

In general, all Japanese citizens younger than 65 years belong to one of four major insurance schemes (i.e., national health insurance [~ 26.6 million members], public-corporation-run health insurance [~ 39.0 million], society-managed, employment-based health insurance [~ 27.6 million], and mutual aid association [~ 9.6 million members]) (Ministry of Health, Labour and Welfare of Japan n.d.). The database used in this study is based on society-managed employment-based health insurance, to which salaried employees of large corporations belong. As the characteristics of individuals of reproductive age in the JMDC database are comparable to those of their counterparts in the national database (Barberio et al. 2024; Nagai et al. 2021) and the insurance scheme depends on the work situation, potential differences in healthcare systems, genetic backgrounds, and environmental factors may be limited, and the findings of this study are considered applicable to other settings.

This study has some limitations. First, not all pregnancies and outcomes were included. In previous validation studies, the sensitivity of the pregnancy estimation algorithm in women who were not linked to their infants was 73.4% (Tajima et al. 2022b), while the sensitivity for birth outcomes remained unknown (Tajima et al. 2022a), raising concerns about misclassification. The dataset covered approximately 14% of the Japanese population, potentially affecting its generalizability. Potential differences in healthcare systems, genetic backgrounds, and environmental factors may be limited. Second, residual confounding factors may have persisted owing to limited data on potential confounders, including disease severity, alcohol consumption, and other lifestyle factors. Proxy variables, such as mental health medication count and substance dependence, as well as general markers of disease burden, were used as confounders to mitigate this effect. Third, exposure was based on dispensation data rather than actual intake, potentially leading to misclassification. However, pregnancy onset was estimated using GA at a specific visit rather than assigning a fixed duration (e.g., 270 days), and dispensing dates were available for approximately 98% of dispensations, including those during hospitalization. Fourth, exposure from as-needed dispensations was not considered in the analysis of dose levels because daily dose information was unavailable for as-needed dispensations. Fifth, analyses stratified by dose and half-life were exploratory and the study was not specifically powered to detect small differences across categories.

In conclusion, we found that benzodiazepine exposure in early pregnancy is modestly associated with the risk of miscarriage. Although associations tend to be stronger with higher dose levels and longer half-life categories, results varied across analyses, suggesting that the findings are sensitive to analytic assumptions and should be interpreted with caution. Our findings may reflect Japan’s unique prescription environment, characterized by the predominant use of short-acting agents.

## Supplementary Information

Below is the link to the electronic supplementary material.

Supplementary file1 (DOCX 102 KB)

## Data Availability

None of the data used in this study will be made publicly available to other researchers in compliance with the JMDC Inc. contract.
