# Correction to: Benzodiazepine exposure during early pregnancy and risk of miscarriage: dose–response, half-life classification, and individual agents

**DOI:** 10.1007/s00737-026-01767-2

**Published:** 2026-09-17

**Authors:** Tomofumi Ishikawa, Takamasa Sakai, Noriyuki Iwama, Ryo Obara, Kei Morishita, Motohiko Adomi, Tadaharu Kunitoki, Aoi Noda, Mami Ishikuro, Saya Kikuchi, Natsuko Kobayashi, Hiroaki Tomita, Masatoshi Saito, Hidekazu Nishigori, Shinichi Kuriyama, Nariyasu Mano, Taku Obara

**Affiliations:** 1 Laboratory of Biomolecule and Pathophysiological Chemistry, Graduate School of Pharmaceutical Sciences, Tohoku University, Sendai, Japan; 2 Drug Informatics, Faculty of Pharmacy, Meijo University, Nagoya, Japan; 3 Center for Maternal and Perinatal Medicine, Tohoku University Hospital, Sendai, Japan; 4 Division of Molecular Epidemiology, Graduate School of Medicine, Tohoku University, Sendai, Japan; 5 Department of Epidemiology, Harvard T.H. Chan School of Public Health, Boston, USA; 6 Division of Clinical Pharmacology and Therapeutics, Graduate School of Pharmaceutical Sciences, Tohoku University, Sendai, Japan; 7 Department of Preventive Medicine and Epidemiology, Tohoku Medical Megabank Organization, Tohoku University, Sendai, Japan; 8 Department of Pharmaceutical Sciences, Tohoku University Hospital, Sendai, Japan; 9 Department of Psychiatry, Graduate School of Medicine, Tohoku University, Sendai, Japan; 10 Department of Obstetrics and Gynecology, Graduate School of Medicine, Tohoku University, Sendai, Japan; 11 Department of Development and Environmental Medicine, Fukushima Medical Center for Children and Women, Graduate School of Medicine, Fukushima Medical University, Fukushima, Japan; 12 International Research Institute of Disaster Science, Tohoku University, Sendai, Japan

**Correction to: Archives of Women's Mental Health (2026) 29:86**

**https://doi.org/10.1007/s00737-026-01720-3**

In the original publication of this article, several numerical errors were identified in the Abstract and Results sections.

In the Abstract, under the Results subsection, the number of matched cases and controls was incorrectly published. The sentence originally read:

"Overall, 44,118 cases were matched with 132,317 controls, with a mean age of 33.2 years."

This has been corrected to:

"Overall, 63,482 cases were matched with 190,383 controls, with a mean age of 33.2 years."

In the Results section, under the first paragraph on page 5, text regarding the adjusted OR ranges and the number of controls for sensitivity analyses was incorrectly published. The paragraph originally read:

"In the first five sensitivity analyses, the findings were not consistently in agreement with those of the main analysis (Tables S7–S11). Adjusted ORs for benzodiazepines ranged from 0.984 to 1.263. Dose-dependent trends were inconsistent across some sensitivity analyses, with adjusted ORs as follows: high-dose, 0.969–1.182; medium-dose, 0.936–1.212; and low-dose, 1.069–1.151. The adjusted ORs for long-, intermediate-, and short-acting benzodiazepines were 1.017–1.421, 1.052–1.141, and 0.960–1.220, respectively. In the sixth sensitivity analysis, limited to women diagnosed with anxiety or sleep disorders, 2564 cases were matched with 76,242 controls; the adjusted OR for any benzodiazepine was 1.118 (95% CI, 1.001–1.248) (Table 4)."

This has been corrected to:

"In the first five sensitivity analyses, the findings were not consistently in agreement with those of the main analysis (Tables S7–S11). Adjusted ORs for benzodiazepines ranged from 0.984 to 1.263. Dose-dependent trends were inconsistent across some sensitivity analyses, with adjusted ORs as follows: high-dose, 0.969–1.182; medium-dose, 0.936–1.404; and low-dose, 1.069–1.151. The adjusted ORs for long-, intermediate-, and short-acting benzodiazepines were 1.017–1.421, 1.024–1.141, and 0.960–1.220, respectively. In the sixth sensitivity analysis, limited to women diagnosed with anxiety or sleep disorders, 2564 cases were matched with 7624 controls; the adjusted OR for any benzodiazepine was 1.118 (95% CI, 1.001–1.248) (Table 4)."

The original article has been corrected.

