# Supplementary material for: Benzodiazepine exposure during early pregnancy and risk of miscarriage: dose–response, half-life classification, and individual agents
Source: Arch Womens Ment Health. 2026 May 23;29(3):86. doi: 10.1007/s00737-026-01720-3 (PMC13198485; doi:10.1007/s00737-026-01720-3)
Supplement: Supplementary file 1 — Supplementary file1 (DOCX 102 KB) [file 737_2026_1720_MOESM1_ESM.docx]

[Estimation of pregnancy and birth outcomes 2](#_Toc222243434)

[Sensitivity analyses 4](#_Toc222243435)

[Table S1. Definitions of exclusion criteria and confounders considered in this study 5](#_Toc222243436)

[Table S2. Administrative codes used to estimate pregnancy and birth outcomes 9](#_Toc222243437)

[Table S3. Administrative codes used to estimate the delivery date of women linked to their children 10](#_Toc222243438)

[Table S4. Benzodiazepines evaluated in this study 12](#_Toc222243439)

[Table S5. Sensitivity analyses 14](#_Toc222243440)

[Table S6. Dose levels of benzodiazepines 16](#_Toc222243441)

[Table S7. Benzodiazepines and the risk of miscarriage – Sensitivity Analysis 1 (days of supply not considered in the exposure definition) 17](#_Toc222243442)

[Table S8. Benzodiazepines and the risk of miscarriage – Sensitivity Analysis 2 (exposure defined as a window between pregnancy onset and 28 days before the index date) 18](#_Toc222243443)

[Table S9. Benzodiazepines and the risk of miscarriage – Sensitivity Analysis 3 (only the first trimester was considered the exposure window) 19](#_Toc222243444)

[Table S10. Benzodiazepines and the risk of miscarriage – Sensitivity Analysis 4 (cases with miscarriage before 12 weeks of gestation and their controls) 20](#_Toc222243445)

[Table S11. Benzodiazepines and the risk of miscarriage – Sensitivity Analysis 5 (outpatient-dispensed benzodiazepines only) 21](#_Toc222243446)

[Figure S1. Estimation of dates of pregnancy onset and delivery 22](#_Toc222243447)

Estimation of pregnancy and birth outcomes

Two approaches were applied to estimate pregnancy and birth outcomes (Figure 1). The first pattern was applied to individuals whose pregnancy and birth outcome-related entries (i.e., diagnoses, procedures, or medication information), from which pregnancies and birth outcomes were estimated (Figure S1). Pregnancy was estimated based on validated entries (Table S2), with a positive predictive value (PPV) of 99.8% and sensitivity of 73.4% (Tajima et al. 2022b). The earliest birth outcomes in the database were estimated using validated algorithms (Table S2), including miscarriage, induced abortion, and live birth, with PPVs of 100.0%, 89.2%, and 98.1%, respectively (sensitivity not reported) (Tajima et al. 2022a). Stillbirth was not validated owing to the limited sample size in the validation study (Tajima et al. 2022a) and therefore could not be estimated. In the second pattern, pregnancies were classified as live births if the infants were enrolled under the same insurance. The delivery date was estimated from delivery-related entries (Table S3) or, if unavailable, from the infant’s birth month (i.e., the 15^th^ day of the neonatal birth month) (Figure S1) (Ishikawa et al. 2018; Ishikawa et al. 2019; Ishikawa et al. 2023; Ishikawa et al. 2024; Ishikawa et al. 2025). In administrative data from a university hospital, 96.4% of estimated delivery dates were within ±7 days of the gold standard (Ishikawa et al. 2018).

Pregnancy onset was estimated by subtracting the GA recorded in the diagnosis entries at a specific visit from the diagnosis date (Figure S1). For example, when “iron deficiency anemia at week 30” was entered, 30 weeks and 0 days were subtracted. If multiple GA entries existed, the longest GA was used, as the GA at delivery represents the best obstetric estimate for clinical care (Committee on Obstetric Practice 2017; Japan Society of Obstetrics and Gynecology 2023). Therefore, GA is likely more accurate in the later stages of pregnancy. According to a previous report, 92.8% of estimated onset dates for pregnancy that ended with delivery were within 7 days of the gold standard (Ishikawa et al. 2018).

Sensitivity analyses

The first sensitivity analysis classified pregnancy as exposed only if at least one dispensation occurred between pregnancy onset and the index date (Huybrechts, 2019). The second analysis considered the pregnancy as exposed when supply days overlapped the window between pregnancy onset and 28 days before the index date, accounting for the lag time between developmental arrest and miscarriage (Sundermann et al. 2019; van Gelder et al. 2023). The third analysis considered the pregnancy as exposed if the supply days overlapped with the first trimester (i.e., pregnancy onset through 14 weeks of gestation or the index date, whichever occurred earlier). The fourth analysis restricted the population to cases that resulted in miscarriages before 12 weeks of gestation and their controls to mitigate longitudinal changes in status during pregnancy. The fifth analysis included only outpatient dispensations of benzodiazepines to minimize confounding and reverse causal bias. The sixth analysis limited the population to women diagnosed with anxiety or sleep disorders and repeated the same analyses after risk-set sampling with replacement in the alternative population. The first four sensitivity analyses were also applied to dose and half-life categories, as well as major benzodiazepines.

| Table S1. Definitions of exclusion criteria and confounders considered in this study | | |
| --- | --- | --- |
|  | **Definition** | **Rationale** |
| **Diagnoses and medications excluded from the study population^a,b^** | | |
| Recurrent pregnancy loss | ICD-10 code N96, O262, or standardized disease code 7599016 (recurrent pregnancy loss); dispensation of low-dose aspirin (100 or 81 mg tablets) or heparin calcium (subcutaneous injection syringe) | Excluded because recurrent pregnancy loss is a known risk factor for miscarriage^1^ and may introduce strong baseline risk (confounding) |
| Antiphospholipid syndrome | ICD-10 code D686, O991, or standardized disease code 8841124 (lupus thrombocytopenia) | Excluded because antiphospholipid syndrome is a known risk factor for miscarriage^1^ and may introduce strong baseline risk (confounding) |
| Medications that potentially pose a risk of miscarriage | Thalidomide, pomalidomide, lenalidomide, mycophenolate mofetil, methotrexate, misoprostol, or warfarin | Excluded because these medications have a suspected risk of miscarriage according to the clinical practice guidelines^2^ and could strongly confound the association |
| Epilepsy | ICD-10 code G40 or G41 | Excluded because epilepsy and its treatment may be associated with adverse pregnancy outcomes^3^, and benzodiazepines are also prescribed for epilepsy; this study focused on benzodiazepines prescribed for anxiety, insomnia, and mood disorders |
| **Main indications for benzodiazepines or other psychiatric disorders^c^** | | |
| Anxiety disorder | ICD-10 code F40–F43 | Therapeutic indication of benzodiazepines or other psychiatric medical conditions |
| Sleep disorder | ICD-10 code G47, F51 |  |
| Schizophrenia | ICD-10 code F20 |  |
| Manic episode | ICD-10 code F30 |  |
| Bipolar affective disorder | ICD-10 code F31 |  |
| Depressive disorder | ICD-10 code F32, F33 |  |
| **Uterine diseases^c^** | | |
| Endometriosis | ICD-10 code N80 | Known risk factor for miscarriage^1^ |
| Polyp of corpus uteri | ICD-10 code N840 | Known risk factor for miscarriage^1^ |
| Polyp of cervix uteri | ICD-10 code N841 | Known risk factor for miscarriage^1^ |
| Cervix carcinoma | ICD-10 code C53 | Known risk factor for miscarriage^1^ |
| Corpus uteri carcinoma | ICD-10 code C54 |  |
| Congenital uterus and cervix malformations | ICD-10 code Q51 | Known risk factor for miscarriage^1^ |
| **Other maternal comorbidities as a risk factor for miscarriage^c^** | | |
| Polycystic ovary syndrome | ICD-10 code E282 | Known risk factor for miscarriage^1^ |
| Diabetes | ICD-10 code E10–E14, O24 | Known risk factor for miscarriage^1^ |
| Obesity | ICD-10 code E66 | Known risk factor for miscarriage^1^ |
| Thyroid disorder | ICD-10 code E00–E07 | Known risk factor for miscarriage^1^ |
| **Dependence^c^** | | |
| Alcohol | ICD-10 code F10, Z714, Z721 | Known risk factor for miscarriage^1^ |
| Tobacco | ICD-10 code F17, Z716, Z720  Standardized disease code 8843945 (nicotine dependence during pregnancy) | Known risk factor for miscarriage^1^ |
| **Factors associated with disease severity or burden** | | |
| No. of mental health medications dispensed in the 6 months prior to pregnancy onset | No. of distinct mental health drugs with the following WHO-ATC codes, counted based on generic names dispensed within 180 days prior to pregnancy onset  WHO-ATC code: N05A, N05B, N05C and N06A, excluding benzodiazepines listed in Table S4 | Proxy for severity of mental health disorder |
| No. of other medications dispensed in the 6 months prior to pregnancy onset | No. of distinct drugs, with counting based on generic names dispensed within 180 days prior to pregnancy onset, excluding benzodiazepines listed in Table S4, and other mental health medications as defined above | General markers of illness burden^4^ |
| No. of diagnoses in the 6 months prior to pregnancy onset | No. of distinct diagnoses based on ICD-10 blocks (e.g., A00–A09) within 6 months prior to pregnancy onset (i.e., in the month of pregnancy onset and the preceding 5 months), excluding diagnoses included in other covariates (i.e., indications for benzodiazepines or other psychiatric disorders, uterine diseases, other maternal comorbidities and dependence as a risk factor for miscarriage) | General markers of illness burden^4^ |
| Abbreviations: ATC = Anatomical Therapeutic Chemical, ICD-10: International Classification of Diseases, NA: not applicable, WHO = World Health Organization.  **^a^** Diagnoses before 22 weeks of gestation or delivery, whichever came first (including pre-pregnancy), based on the year and month of the claim were considered unless otherwise specified.  ^b^ Medications dispensed between the pregnancy onset date and 22 weeks of gestation or delivery, whichever came first, were considered unless otherwise specified  ^c^ Diagnoses within 6 months prior to pregnancy onset through the index date based on the year and month of the claim were considered unless otherwise specified.  References:  1. Quenby S, Gallos ID, Dhillon-Smith RK, et al. Miscarriage matters: the epidemiological, physical, psychological, and economic costs of early pregnancy loss. Lancet 2021;397(10285):1658–1667.  2. Japan Society of Obstetrics and Gynecology and Japan Association of Obstetricians and Gynecologists. Guidelines for obstetrical practice in Japan 2023 edition. Japan Society of Obstetrics and Gynecology; 2023 (in Japanese).  3. Oliveira CI, Fett-Conte AC. Birth defects: risk factors and consequences. J Pediatr Genet 2013;2(2):85–90.  4. Huybrechts KF, Bateman BT, Hernández-Díaz S. Use of real-world evidence from healthcare utilization data to evaluate drug safety during pregnancy. Pharmacoepidemiol Drug Saf 2019;28(7):906–922. | | |

| Table S2. Administrative codes used to estimate pregnancy and birth outcomes | | |
| --- | --- | --- |
|  |  | **Codes** |
| Pregnancy | Diagnosis | ICD-10 code: O008, O009, O010, O011, O021, O028, O034, O039, O049, O080, O081, O11, O121, O13, O140, O141, O142, O149, O16, O200, O210, O211, O234, O235, O240, O241, O244, O300, O321, O322, O331, O339, O341, O342, O348, O359, O360, O361, O364, O365, O366, O40, O410, O411, O420, O421, O429, O441, O450, O459, O470, O471, O479, O48, O600, O601, O624, O640, O654, O655, O669, O680, O683, O690 O700 O711, O717, O720, O721, O723, O757, O800, O820, O85, O860, O863, O872, O881, O908, O911, O926, O983, O984, O990, O991, O996, P022, P059, P209, P551, P832, P95, N853 |
|  | Procedure | Procedure code: A205-3, A236-2, A237, A303, D218, D219, J071, J077, J080, K893, K896, K898, K902, K903, K904, K906, K909, K909-2, K911 |
|  | Medication | WHO-ATC code: G02AB01 (injection, tablet), G02AD01 (injection), G02AD03 (topical medication), H01BB02 (injection) |
| Miscarriage | Diagnosis | ICD-10 code: O008, O010, O034, O039, O080, P95 |
|  | Procedure | Procedure code: K909, K911 |
|  | Medication | N/A |
| Induced abortion | Diagnosis | ICD-10 code: O049 |
|  | Procedure | Procedure code: K909-2 |
|  | Medication | N/A |
| Live birth | Diagnosis | ICD-10 code: O601, O624, O654, O655, O669, O683, O690, O700, O717, O721, O800 |
|  | Procedure | Procedure code: K893, K896, K898, K904 |
|  | Medication | WHO-ATC code: G02AD01 (injection), H01BB02 (injection) |
| Abbreviations: ATC = Anatomical Therapeutic Chemical, ICD-10 = International Classification of Diseases, N/A = not applicable, WHO = World Health Organization. | | |

| Table S3. Administrative codes used to estimate the delivery date of women linked to their children | |
| --- | --- |
| **Diagnosis^a^** | **ICD-10 code** |
| Complications of labor and delivery | O60-O75, excluding O600 (preterm labor without delivery)  (prioritized diagnoses^b^: O601, O602, O603, O622, O629, O640, O655, O669, O683, O690, O709, O711, O717, O721, O723, O730) |
| Delivery | O80-O84 (prioritized diagnoses^b^: O800, O814, O821) |
| **Surgical procedure^c^** | **Procedure code** |
| Cervical incision during delivery (including suturing) | K891 |
| Breech extraction | K892 |
| Vacuum extraction delivery | K893^b^ |
| Forceps delivery | K894 |
| Episiotomy and suturing (during delivery) | K895 |
| Perineal laceration suturing (during delivery) | K896 |
| Cervical laceration suturing (during delivery) | K897 |
| Cesarean section | K898^b^ |
| Umbilical cord reduction surgery | K900 |
| Prolapsed limb reduction surgery | K900-2 |
| Bimanual uterine compression  (including aortic compression) | K901 |
| Manual placental separation | K902 |
| Uterine rupture surgery | K903 |
| Obstetric hysterectomy (Porro operation) | K904^b^ |
| Uterine inversion correction surgery   (vaginal, abdominal) | K905 |
| **Medical procedure^c^** | **Procedure code** |
| Uterine dilation and labor induction | J080 |
| **Dispensed injectable medication^d^** |  |
| Oxytocin | Identified based on the non-proprietary name and route of administration |
| Dinoprost |  |
| Methylergometrine maleate |  |
| Abbreviations: ICD-10, International Classification of Diseases 10th Revision.  ^a^ Date of diagnosis was considered as a delivery date.  ^b^ Prioritized diagnoses or surgical procedures.  ^c^ Implementation date was considered as a delivery date. For K893, K898, and K904 only, in rare circumstances where the implementation date was missing and the hospitalization date was available, the hospitalization date was used to estimate the delivery date.  ^d^ Dispensing date was used to estimate the delivery date. | |

| Table S4. Benzodiazepines evaluated in this study | | | |
| --- | --- | --- | --- |
|  | **WHO-ATC codes** | **Half-life categories** | **Dose equivalence** |
| Chlordiazepoxide | N05BA02 | Long-acting | 10 |
| Clonazepam | N03AE01 | Long-acting | 0.25 |
| Clorazepate | N05BA05 | Long-acting | 7.5 |
| Cloxazolam | N05BA22 | Long-acting | 1.5 |
| Diazepam  (oral only) | N05BA01 (suppositories are excluded) | Long-acting | 5 |
| Fludiazepam | N05BA17 | Long-acting | 0.5 |
| Flurazepam | N05CD01 | Long-acting | 15 |
| Flutoprazepam | N05BA^a^ | Long-acting | 1.67 |
| Haloxazolam | N05CD^a^ | Long-acting | 5 |
| Loflazepate | N05BA18 | Long-acting | 1.67 |
| Medazepam | N05BA03 | Long-acting | 10 |
| Mexazolam | N05BA25 | Long-acting | 1.67 |
| Oxazolam | N05BA^a^ | Long-acting | 20 |
| Prazepam | N05BA11 | Long-acting | 12.5 |
| Quazepam | N05CD10 | Long-acting | 15 |
| Alprazolam | N05BA12 | Intermediate-acting | 0.8 |
| Bromazepam | N05BA08 | Intermediate-acting | 2.5 |
| Estazolam | N05CD04 | Intermediate-acting | 2 |
| Flunitrazepam | N05CD03 (injections are excluded) | Intermediate-acting | 1 |
| Lorazepam | N05BA06 (injections are excluded) | Intermediate-acting | 1.2 |
| Nimetazepam | N05CD15 | Intermediate-acting | 5 |
| Nitrazepam | N05CD02 | Intermediate-acting | 5 |
| Brotizolam | N05CD09 | Short-acting | 0.25 |
| Clotiazepam | N05BA21 | Short-acting | 10 |
| Etizolam | N05BA19 | Short-acting | 1.5 |
| Flutazolam | N05BA^a^ | Short-acting | 15 |
| Lormetazepam | N05CD06 | Short-acting | 1 |
| Tofisopam | N05BA23 | Short-acting | 125 |
| Triazolam | N05CD05 | Short-acting | 0.25 |
| Rilmazafone | N05CD^a^ | Short-acting | 2 |
| Abbreviations: ATC = Anatomical Therapeutic Chemical, WHO = World Health Organization.  Some substances of the thienodiazepine class are included.  Non-oral benzodiazepines (i.e., bromazepam suppository, diazepam injection, diazepam suppository, flunitrazepam injection, lorazepam injection, remimazolam injection, midazolam injection, midazolam oromucosal solution) or oral benzodiazepines indicated generally for epilepsy (i.e., clobazam) were not within the scope of this study.  ^a^ The specific WHO-ATC codes are unavailable for these drugs; identification was based on non-proprietary names. | | | |

| Table S5. Sensitivity analyses | | |
| --- | --- | --- |
|  | **Cases and controls** | **Definition of exposure** |
| Main analysis  (Table 3) | All cases and their controls | Considered exposed if:   - A mother had a dispensation from pregnancy onset to the day before the index date - The days of supply overlapped the window between pregnancy onset and the day before the index date |
| Sensitivity analysis #1 (Table S7) | Same as the main analysis | Considered exposed if:   - A mother had a dispensation from pregnancy onset until the day before the index date - **No. of days of supply was not considered** |
| Sensitivity analysis #2 (Table S8) | Same as the main analysis | Considered exposed if:   - A mother had a dispensation from pregnancy onset to **28 days before the index date** - The days of supply overlapped the window between pregnancy onset and **28 days before the index date** |
| Sensitivity analysis #3  (Table S9) | Same as the main analysis | Considered exposed if:   - A mother had a dispensation from pregnancy onset to **the end of the first trimester (i.e., 14 weeks of gestation)** - The days of supply overlapped the window between pregnancy onset and **the end of the first trimester (i.e., 14 weeks of gestation)** |
| Sensitivity analysis #4  (Table S10) | **Cases with miscarriage before 12 weeks of gestation and their controls** | Same as the main analysis |
| Sensitivity analysis #5  (Table S11) | Same as the main analysis | Considered exposed if:   - A mother had **an outpatient** dispensation from pregnancy onset to the day before the index date - The days of supply for **outpatient** dispensations overlapped the window between pregnancy onset and the day before the index date |
| Sensitivity analysis #6  (Table 4) | **Cases and controls diagnosed with anxiety or sleep disorders** | Same as the main analysis |

| Table S6. Dose levels of benzodiazepines | | | | | | |
| --- | --- | --- | --- | --- | --- | --- |
|  | **Cases n = 63,482** | | | **Controls n = 190,383** | | |
|  | **High-dose** | **Medium-dose** | **Low-dose** | **High-dose** | **Medium-dose** | **Low-dose** |
| Long-acting | 98 (0.2) | 130 (0.2) | 54 (0.1) | 186 (0.1) | 245 (0.1) | 114 (0.1) |
| Diazepam | 25 (0.0) | 51 (0.1) | 13 (0.0) | 48 (0.0) | 31 (0.0) | 22 (0.0) |
| Loflazepate | 29 (0.0) | 58 (0.1) | 37 (0.1) | 53 (0.0) | 153 (0.1) | 78 (0.0) |
| Intermediate-acting | 197 (0.3) | 132 (0.2) | 65 (0.1) | 397 (0.2) | 354 (0.2) | 132 (0.1) |
| Alprazolam | 64 (0.1) | 59 (0.1) | 26 (0.0) | 168 (0.1) | 169 (0.1) | 70 (0.0) |
| Bromazepam | 37 (0.1) | 20 (0.0) | 4 (0.0) | 89 (0.0) | 37 (0.0) | 15 (0.0) |
| Flunitrazepam | 58 (0.1) | 16 (0.0) | 7 (0.0) | 118 (0.1) | 44 (0.0) | 20 (0.0) |
| Lorazepam | 37 (0.1) | 35 (0.1) | 23 (0.0) | 70 (0.0) | 77 (0.0) | 27 (0.0) |
| Nitrazepam | 34 (0.1) | 8 (0.0) | 4 (0.0) | 35 (0.0) | 30 (0.0) | 5 (0.0) |
| Short-acting | 190 (0.3) | 166 (0.3) | 108 (0.2) | 386 (0.2) | 344 (0.2) | 273 (0.1) |
| Brotizolam | 92 (0.1) | 28 (0.0) | 21 (0.0) | 143 (0.1) | 94 (0.0) | 45 (0.0) |
| Clotiazepam | 32 (0.1) | 37 (0.1) | 19 (0.0) | 79 (0.0) | 81 (0.0) | 41 (0.0) |
| Etizolam | 56 (0.1) | 77 (0.1) | 64 (0.1) | 141 (0.1) | 146 (0.1) | 174 (0.1) |
| Triazolam | 28 (0.0) | 15 (0.0) | 3 (0.0) | 67 (0.0) | 28 (0.0) | 6 (0.0) |
| Benzodiazepines with a prevalence of ≥0.1% in either the case or control group. | | | | | | |

| Table S7. Benzodiazepines and the risk of miscarriage – Sensitivity Analysis 1 (days of supply not considered in the exposure definition) | | |
| --- | --- | --- |
|  | **Crude OR**  **(95% CI)** | **Adjusted OR^a^ (95% CI)** |
| Any benzodiazepine | 1.421 (1.315, 1.537) | 1.263 (1.150, 1.387) |
| Classified by dose level | | |
| High-dose | 1.358 (1.204, 1.531) | 1.124 (0.982 1.286) |
| Medium-dose | 1.570 (1.360, 1.812) | 1.404 (1.201, 1.642) |
| Low-dose | 1.247 (1.007, 1.543) | 1.069 (0.852, 1.340) |
| Classified by half-life and individual benzodiazepine | | |
| Long-acting | 1.699 (1.465, 1.971) | 1.421 (1.209, 1.670) |
| Diazepam | 3.033 (2.270, 4.052) | 2.327 (1.713, 3.162) |
| Loflazepate | 1.379 (1.105, 1.721) | 1.220 (0.964, 1.545) |
| Intermediate-acting | 1.261 (1.120, 1.419) | 1.070 (0.937, 1.223) |
| Alprazolam | 0.984 (0.816, 1.187) | 0.823 (0.674, 1.005) |
| Bromazepam | 1.252 (0.921, 1.701) | 0.985 (0.713, 1.361) |
| Flunitrazepam | 1.215 (0.920, 1.604) | 1.088 (0.810, 1.460) |
| Lorazepam | 1.568 (1.235, 1.991) | 1.275 (0.987, 1.646) |
| Nitrazepam | 2.344 (1.619, 3.393) | 2.146 (1.442, 3.193) |
| Short-acting | 1.436 (1.286, 1.603) | 1.220 (1.078, 1.380) |
| Brotizolam | 1.629 (1.328, 1.998) | 1.525 (1.222, 1.902) |
| Clotiazepam | 1.311 (1.021, 1.682) | 1.133 (0.869, 1.477) |
| Etizolam | 1.248 (1.054, 1.477) | 0.963 (0.803, 1.156) |
| Triazolam | 1.404 (0.982, 2.009) | 1.160 (0.797, 1.688) |
| Abbreviations: CI = confidence interval, OR = odds ratio  ^a^ Adjusted for the main indications for benzodiazepines (anxiety disorder or sleep disorder); other psychiatric disorders (schizophrenia, manic episode, bipolar affective disorder, or depressive disorder); uterine diseases (endometriosis, polyp of corpus uteri or cervix uteri, cervix or corpus uteri carcinoma, or congenital uterus and cervix malformations); other maternal comorbidities (polycystic ovary syndrome, diabetes, obesity, or thyroid disorders); alcohol or tobacco dependence; the number of mental health medications dispensed in the 6 months prior to pregnancy onset; the number of other medications dispensed in the 6 months prior to pregnancy onset; and the number of diagnoses in the 6 months prior to pregnancy onset. | | |

| Table S8. Benzodiazepines and the risk of miscarriage – Sensitivity Analysis 2 (exposure defined as a window between pregnancy onset and 28 days before the index date) | | |
| --- | --- | --- |
|  | **Crude OR**  **(95% CI)** | **Adjusted OR^a^ (95% CI)** |
| Any benzodiazepine | 1.200 (1.106, 1.302) | 0.984 (0.892, 1.086) |
| Classified by dose level | | |
| High-dose | 1.221 (1.063, 1.403) | 0.976 (0.836, 1.139) |
| Medium-dose | 1.116 (0.966, 1.290) | 0.936 (0.799, 1.095) |
| Low-dose | 1.341 (1.132, 1.588) | 1.101 (0.918, 1.321) |
| Classified by half-life and individual benzodiazepine | | |
| Long-acting | 1.267 (1.082, 1.483) | 1.017 (0.856, 1.208) |
| Diazepam | 1.319 (0.909, 1.913) | 0.957 (0.645, 1.418) |
| Loflazepate | 1.270 (1.022, 1.578) | 1.083 (0.860, 1.365) |
| Intermediate-acting | 1.261 (1.117, 1.424) | 1.052 (0.918, 1.206) |
| Alprazolam | 1.045 (0.862, 1.267) | 0.860 (0.700, 1.056) |
| Bromazepam | 1.362 (1.001, 1.852) | 1.063 (0.767, 1.474) |
| Flunitrazepam | 1.272 (0.971, 1.667) | 1.147 (0.861, 1.528) |
| Lorazepam | 1.631 (1.285, 2.071) | 1.333 (1.032, 1.722) |
| Nitrazepam | 1.091 (0.684, 1.741) | 0.951 (0.571, 1.582) |
| Short-acting | 1.193 (1.063, 1.339) | 0.960 (0.844, 1.092) |
| Brotizolam | 1.217 (0.974, 1.521) | 1.103 (0.867, 1.404) |
| Clotiazepam | 1.255 (0.977, 1.613) | 1.043 (0.799, 1.361) |
| Etizolam | 1.142 (0.963, 1.355) | 0.869 (0.723, 1.045) |
| Triazolam | 1.245 (0.857, 1.808) | 0.992 (0.671, 1.466) |
| Abbreviations: CI = confidence interval, OR = odds ratio  ^a^ Adjusted for the main indications for benzodiazepines (anxiety disorder or sleep disorder); other psychiatric disorders (schizophrenia, manic episode, bipolar affective disorder, or depressive disorder); uterine diseases (endometriosis, polyp of corpus uteri or cervix uteri, cervix or corpus uteri carcinoma, or congenital uterus and cervix malformations); other maternal comorbidities (polycystic ovary syndrome, diabetes, obesity, or thyroid disorders); alcohol or tobacco dependence; the number of mental health medications dispensed in the 6 months prior to pregnancy onset; the number of other medications dispensed in the 6 months prior to pregnancy onset; and the number of diagnoses in the 6 months prior to pregnancy onset. | | |

| Table S9. Benzodiazepines and the risk of miscarriage – Sensitivity Analysis 3 (only the first trimester was considered the exposure window) | | |
| --- | --- | --- |
|  | **Crude OR**  **(95% CI)** | **Adjusted OR^a^ (95% CI)** |
| Any benzodiazepine | 1.393 (1.294, 1.499) | 1.211 (1.107, 1.324) |
| Classified by dose level | | |
| High-dose | 1.440 (1.264, 1.640) | 1.182 (1.022, 1.366) |
| Medium-dose | 1.392 (1.229, 1.576) | 1.187 (1.035, 1.362) |
| Low-dose | 1.326 (1.128, 1.558) | 1.106 (0.930, 1.316) |
| Classified by half-life and individual benzodiazepine | | |
| Long-acting | 1.550 (1.348, 1.783) | 1.277 (1.096, 1.488) |
| Diazepam | 2.549 (1.942, 3.346) | 1.971 (1.478, 2.627) |
| Loflazepate | 1.302 (1.058, 1.602) | 1.109 (0.889, 1.383) |
| Intermediate-acting | 1.322 (1.183, 1.478) | 1.113 (0.981, 1.263) |
| Alprazolam | 1.048 (0.879, 1.248) | 0.853 (0.707, 1.030) |
| Bromazepam | 1.349 (1.011, 1.800) | 1.062 (0.782, 1.441) |
| Flunitrazepam | 1.341 (1.037, 1.736) | 1.224 (0.931, 1.609) |
| Lorazepam | 1.641 (1.316, 2.047) | 1.327 (1.046, 1.682) |
| Nitrazepam | 2.057 (1.425, 2.970) | 1.891 (1.273, 2.809) |
| Short-acting | 1.375 (1.239, 1.526) | 1.141 (1.015, 1.283) |
| Brotizolam | 1.542 (1.270, 1.872) | 1.430 (1.160, 1.764) |
| Clotiazepam | 1.278 (1.018, 1.605) | 1.068 (0.838, 1.360) |
| Etizolam | 1.214 (1.035, 1.422) | 0.929 (0.782, 1.103) |
| Triazolam | 1.366 (0.964, 1.936) | 1.108 (0.769, 1.596) |
| Abbreviations: CI = confidence interval, OR = odds ratio  ^a^ Adjusted for the main indications for benzodiazepines (anxiety disorder or sleep disorder); other psychiatric disorders (schizophrenia, manic episode, bipolar affective disorder, or depressive disorder); uterine diseases (endometriosis, polyp of corpus uteri or cervix uteri, cervix or corpus uteri carcinoma, or congenital uterus and cervix malformations); other maternal comorbidities (polycystic ovary syndrome, diabetes, obesity, or thyroid disorders); alcohol or tobacco dependence; the number of mental health medications dispensed in the 6 months prior to pregnancy onset; the number of other medications dispensed in the 6 months prior to pregnancy onset; and the number of diagnoses in the 6 months prior to pregnancy onset. | | |

| Table S10. Benzodiazepines and the risk of miscarriage – Sensitivity Analysis 4 (cases with miscarriage before 12 weeks of gestation and their controls) | | |
| --- | --- | --- |
|  | **Crude OR**  **(95% CI)** | **Adjusted OR^a^ (95% CI)** |
| Any benzodiazepine | 1.393 (1.293, 1.500) | 1.238 (1.130, 1.355) |
| Classified by dose level | | |
| High-dose | 1.400 (1.225, 1.600) | 1.165 (1.005, 1.351) |
| Medium-dose | 1.397 (1.232, 1.584) | 1.212 (1.055, 1.392) |
| Low-dose | 1.351 (1.148, 1.589) | 1.151 (0.967, 1.371) |
| Classified by half-life and individual benzodiazepine | | |
| Long-acting | 1.554 (1.349, 1.789) | 1.295 (1.110, 1.511) |
| Diazepam | 2.541 (1.930, 3.344) | 1.988 (1.488, 2.658) |
| Loflazepate | 1.303 (1.057, 1.607) | 1.125 (0.900, 1.406) |
| Intermediate-acting | 1.323 (1.182, 1.481) | 1.141 (1.004, 1.296) |
| Alprazolam | 1.073 (0.899, 1.279) | 0.896 (0.742, 1.082) |
| Bromazepam | 1.331 (0.989, 1.791) | 1.040 (0.760, 1.423) |
| Flunitrazepam | 1.322 (1.017, 1.717) | 1.216 (0.921, 1.606) |
| Lorazepam | 1.639 (1.309, 2.051) | 1.362 (1.071, 1.734) |
| Nitrazepam | 2.014 (1.392, 2.915) | 1.912 (1.287, 2.841) |
| Short-acting | 1.358 (1.222, 1.509) | 1.149 (1.020, 1.294) |
| Brotizolam | 1.472 (1.206, 1.795) | 1.394 (1.125, 1.727) |
| Clotiazepam | 1.282 (1.016, 1.617) | 1.096 (0.856, 1.402) |
| Etizolam | 1.211 (1.031, 1.423) | 0.948 (0.796, 1.128) |
| Triazolam | 1.273 (0.887, 1.826) | 1.070 (0.734, 1.560) |
| Abbreviations: CI = confidence interval, OR = odds ratio  ^a^ Adjusted for the main indications for benzodiazepines (anxiety disorder or sleep disorder); other psychiatric disorders (schizophrenia, manic episode, bipolar affective disorder, or depressive disorder); uterine diseases (endometriosis, polyp of corpus uteri or cervix uteri, cervix or corpus uteri carcinoma, or congenital uterus and cervix malformations); other maternal comorbidities (polycystic ovary syndrome, diabetes, obesity, or thyroid disorders); alcohol or tobacco dependence; the number of mental health medications dispensed in the 6 months prior to pregnancy onset; the number of other medications dispensed in the 6 months prior to pregnancy onset; and the number of diagnoses in the 6 months prior to pregnancy onset. | | |

| Table S11. Benzodiazepines and the risk of miscarriage – Sensitivity Analysis 5 (outpatient-dispensed benzodiazepines only) | | |
| --- | --- | --- |
|  | **Crude OR**  **(95% CI)** | **Adjusted OR^a^ (95% CI)** |
| Any benzodiazepine | 1.260 (1.167, 1.360) | 1.058 (0.965, 1.161) |
| Classified by dose level | | |
| High-dose | 1.217 (1.059, 1.398) | 0.969 (0.831, 1.131) |
| Medium-dose | 1.329 (1.172, 1.508) | 1.127 (0.981, 1.295) |
| Low-dose | 1.337 (1.138, 1.572) | 1.125 (0.945, 1.338) |
| Classified by half-life and individual benzodiazepine | | |
| Long-acting | 1.482 (1.285, 1.708) | 1.220 (1.044, 1.425) |
| Diazepam | 2.216 (1.666, 2.948) | 1.735 (1.283, 2.346) |
| Loflazepate | 1.312 (1.067, 1.613) | 1.115 (0.894, 1.390) |
| Intermediate-acting | 1.231 (1.099, 1.380) | 1.024 (0.900, 1.166) |
| Alprazolam | 1.025 (0.858, 1.224) | 0.837 (0.692, 1.012) |
| Bromazepam | 1.340 (1.005, 1.787) | 1.060 (0.781, 1.438) |
| Flunitrazepam | 1.245 (0.956, 1.622) | 1.142 (0.863, 1.511) |
| Lorazepam | 1.608 (1.286, 2.009) | 1.310 (1.032, 1.664) |
| Nitrazepam | 1.141 (0.732, 1.777) | 0.955 (0.590, 1.547) |
| Short-acting | 1.224 (1.098, 1.364) | 0.995 (0.880, 1.125) |
| Brotizolam | 1.224 (0.989, 1.515) | 1.136 (0.902, 1.431) |
| Clotiazepam | 1.254 (0.997, 1.577) | 1.049 (0.822, 1.338) |
| Etizolam | 1.204 (1.027, 1.413) | 0.920 (0.774, 1.093) |
| Triazolam | 1.366 (0.964, 1.936) | 1.108 (0.769, 1.596) |
| Abbreviations: CI = confidence interval, OR = odds ratio  ^a^ Adjusted for main indications of benzodiazepines (anxiety disorder or sleep disorder), other psychiatric disorders (schizophrenia, manic episode, bipolar affective disorder, depressive disorder), uterine diseases (endometriosis, polyp of corpus uteri or cervix uteri, cervix or corpus uteri carcinoma, or congenital uterus and cervix malformations), other maternal comorbidities (polycystic ovary syndrome, diabetes, obesity, or thyroid disorders), alcohol or tobacco dependence, number of mental health medications dispensed in the 6 months prior to pregnancy onset, number of other medications dispensed in the 6 months prior to pregnancy onset, and number of diagnoses in the 6 months prior to pregnancy onset. | | |

Figure S1. Estimation of dates of pregnancy onset and delivery


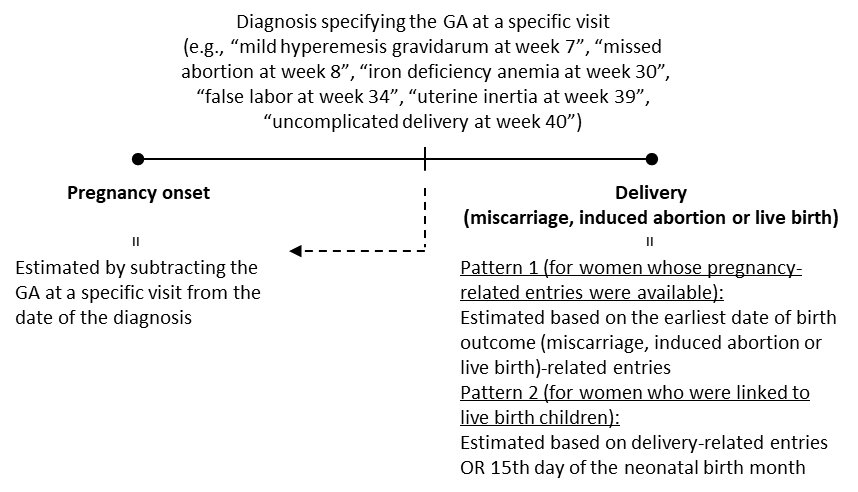


Abbreviations: GA, gestational age.
